# Advancing Colorimetric Analysis in Enzyme-Linked Immunosorbent Assays: Harnessing Nonlinear Regression for Improved Accuracy and Predictive Performance

**DOI:** 10.1021/acsomega.5c10509

**Published:** 2026-04-22

**Authors:** Shaghayegh Mirhosseini, Aryanaz Faghih Nasiri, Fatemeh Khatami, Akram Mirzaei, Seyed Mohammad Kazem Aghamir, Nathan S. Swami, Mohammadreza Kolahdouz

**Affiliations:** † School of Electrical and Computer Engineering, College of Engineering, 48425 University of Tehran, Tehran 1439957131, Iran; ‡ Urology Research Center, 48439 Tehran University of Medical Sciences, Tehran 1416753955, Iran; § Department of Electrical and Computer Engineering, 2358 University of Virginia, Charlottesville, Virginia 22904, United States; ∥ Chemistry, University of Virginia, Charlottesville, Virginia 22904, United States

## Abstract

Smartphone-based colorimetric enzyme-linked immunosorbent assay (ELISA) readers have emerged as a cost-effective and portable alternative to conventional spectrophotometric systems, especially for use in resource-limited and point-of-care settings. In our previous work, we developed a digital image colorimetry platform integrated with a 3D-printed optomechanical system and smartphone imaging, achieving high diagnostic accuracy for cancer cell lines through linear regression modeling of red, green, and blue (RGB) intensities. While effective, this linear approach could not fully capture the nonlinear relationships intrinsic to enzymatic colorimetric reactions and light–matter interactions. In this study, we present a significant enhancement to our prior model by implementing a nonlinear machine learning framework, eXtreme gradient boosting (XGBoost), to better predict optical densities from smartphone-captured RGB data. Utilizing the same hardware platform and experimental protocol as our earlier system, we extracted RGB values from ELISA images under RGB backlighting and then expanded the feature space through engineered transformations. These features were used to train and validate the XGBoost model on multiple human cancer cell lines (HE4, PC3, 5637, and ACHN). Our XGBoost model achieved exceptional predictive performance with *R* ^2^ > 0.999 across all tested cell lines, substantially improving upon the linear regression model (*R* ^2^ range: 0.923–0.996). Root mean square error was reduced by over 98%, and Spearman and Pearson correlation coefficients approached unity, demonstrating excellent trend fidelity and linear alignment with FDA-certified ELISA readers (Epoch and Tecan). The model’s robustness was further confirmed through k-fold cross-validation and rigorous statistical analysis. This advancement positions our platform as a powerful candidate for decentralized diagnostics and high-throughput screening, capable of operating reliably across different smartphones and lighting conditions.

## Graphical abstract

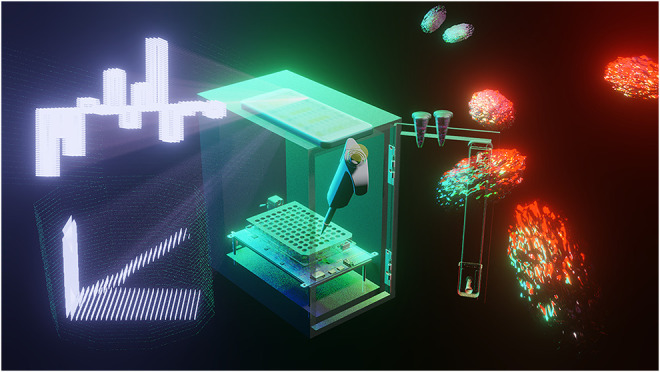

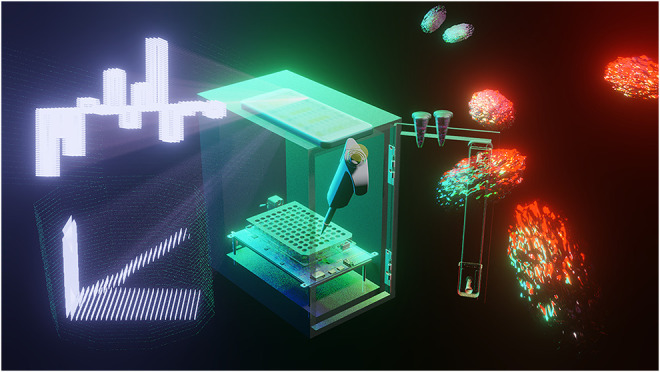

## Introduction

Enzyme-linked immunosorbent assays (ELISAs) are among the most widely adopted analytical techniques for detecting and quantifying specific biomolecules and have become an indispensable tool in clinical diagnostics, pharmaceutical development, food safety testing, environmental monitoring, and diverse biological research.^1–3^ Since their introduction in the 1970s, ELISAs have been valued for their high sensitivity, excellent specificity, and versatility in accommodating a broad range of analytes, from proteins and peptides to hormones, toxins, and pathogens.^1–3^ The method’s success lies in its ability to leverage enzyme-mediated colorimetric reactions, which provide stable and measurable signals even at very low analyte concentrations.^2^

The standard ELISA format, typically implemented in 96-well microplates, enables simultaneous processing of multiple samples, thus increasing throughput and reducing per-sample costs in comparison to single-use or nonbatched tests such as lateral flow assays.^2,4,5^ Moreover, the multiwell plate format has been FDA-approved for numerous diagnostic applications, ensuring regulatory acceptance and facilitating clinical integration.^6^ Nevertheless, the widespread use of ELISA in both high- and low-resource settings is hampered by its dependence on sophisticated optical absorption-based plate reader spectrophotometric instruments that are expensive, bulky, nonportable, and require trained personnel for operation. These constraints limit deployment outside well-equipped laboratories,^7–9^ restricting the potential method for field diagnostics, epidemiological surveys, and rapid point-of-care (POC) decision-making.

Over the past decade, advancements in digital imaging technologies, consumer electronics, and wireless communication have opened new avenues for overcoming these limitations. High-resolution cameras embedded in smartphones and tablets, combined with powerful onboard processors, have enabled the development of compact, low-cost, and user-friendly imaging-based diagnostic tools. By capturing and analyzing the color intensity of ELISA wells, such systems can replace traditional spectrophotometric readers, translating red, green, and blue (RGB) image data into quantitative analyte concentrations.^7–11^ The use of smartphones offers additional benefits, including portability, rapid image acquisition, instant data processing, wireless connectivity for result sharing, and the possibility of remote diagnostics via telemedicine platforms.^10–12^ Importantly, these devices can operate in resource-limited environments without the need for continuous mains power or specialized infrastructure, making them attractive for global health initiatives, outbreak monitoring, and decentralized laboratory testing.

In our previous study, we developed a smartphone-based digital image colorimetry platform integrated with a 3D-printed optomechanical system. The platform captured ELISA well plate images under controlled illumination and applied a linear regression model to correlate RGB intensities with optical densities measured by FDA-approved benchtop ELISA readers.^13^ This system demonstrated high diagnostic accuracy for multiple cancer cell lines and provided a low-cost, reusable, and portable diagnostic tool. However, linear regression inherently assumes a simplified linear relationship between inputs and outputs, potentially overlooking complex nonlinear dependencies arising from enzymatic kinetics, optical scattering, and heterogeneous color development across wells.

Building upon this foundation, this study introduces a significant enhancement by incorporating eXtreme gradient boosting (XGBoost), a powerful nonlinear ensemble learning algorithm. XGBoost can model intricate, nonlinear relationships by iteratively constructing optimized decision trees, thereby capturing subtle variations in colorimetric patterns that linear models might miss.^14^ Additionally, we expand the feature space through engineered transformations of the raw RGB inputs, enabling the model to better exploit hidden patterns and improve prediction robustness.

By integrating XGBoost with our existing hardware platform, we aim to achieve unprecedented prediction accuracy and reliability in smartphone-based ELISA analysis, surpassing the performance of our previous linear model. This advancement not only strengthens the viability of portable, low-cost ELISA readers for diverse diagnostic settings but also lays the groundwork for future applications in multibiomarker detection, automated classification, and adaptable deployment across varying smartphones and environmental conditions.

## Materials and Methods

### 3D-Printed Opto-Mechanical Colorimetric Device

The smartphone-based colorimetric platform is illustrated in Figure 1. This device is held within a 3D printed black box measuring 141 × 222 × 250 mm^3^, ensuring ambient light isolation. A Samsung A71 was utilized to capture the images from a precisely engineered hole on the top surface of the 3D printed box. The samples are in a 96-well microplate held on a custom-fabricated MDF holder incorporating a 2.7 mm black base and mounted above a 7 in. LCD with 1024 × 600, HDMI, IPS panel specifications, used for its wide angular perspective and its high precision. The LCD is driven by a mainboard operating on the Raspbian operation system and connected with the above-mentioned HDMI. The light received by the smartphone’s camera has been passed through the microplate after being modulated by a filter. Subsequently, three images are captured for each plate, corresponding to RGB LCD lights, which are in the visible light wavelength range and subsequently transmitted to a computational server for further processing, which extracts the three-digit (RGB) value for each well. The obtained values are then used for image processing and machine learning stages.^13^

**Figure 1:**
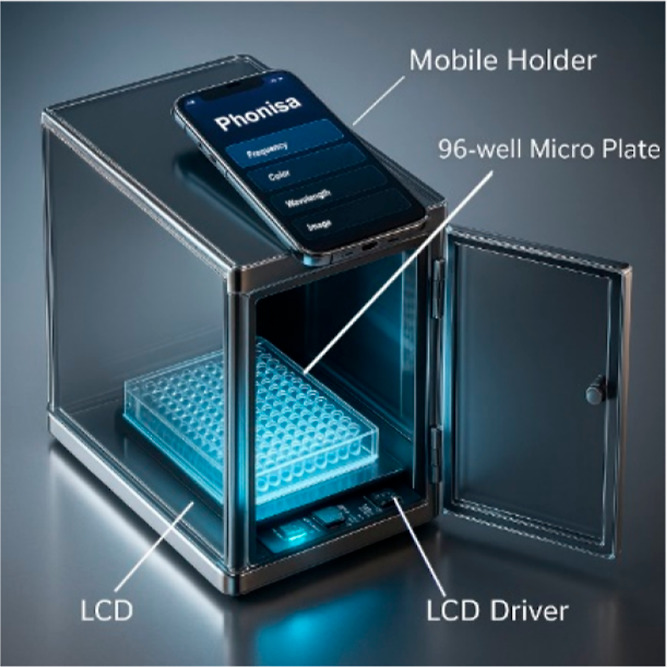
Schematic view and optical density detection for different cancer cells of the cell phone-based ELISA colorimetric reader.

## Reagents and Materials

The study utilized a standard ELISA kit suitable for detecting the target analyte. Key reagents included substrate solutions, washing buffers, and stop solutions, all prepared and handled following the manufacturer’s instructions. Polystyrene 96-well microplates served as the reaction platform. Additionally, a smartphone equipped with a high-resolution camera was employed for image capture, and image-processing software was used for data extraction.

### Sample Preparation and ELISA Procedure

Samples were prepared according to the standard ELISA protocol with minor adjustments to optimize compatibility with the image-based analysis system. Following the coating of microplate wells with the capture antibody, target analyte solutions were added at various concentrations to generate a calibration curve. After incubation, wells were washed to remove unbound substances, and the detection antibody was applied. Subsequently, the substrate solution was introduced, allowing for color development proportional to the analyte concentration. The reaction was terminated using a stop solution, yielding a stable color for analysis.

### Image Capture and Data Processing

After the assay was completed, microplates were placed on a uniformly lit background to minimize variations in lighting conditions during image capture. Images were taken using the smartphone’s camera at a fixed distance and angle to ensure consistency. The images were processed with image-analysis software to extract colorimetric data, specifically RGB intensity values, which were converted into numerical data sets for further analysis.

### Data Analysis

Linear regression analysis was initially performed to establish the relationship between RGB values and the analyte concentration. To explore more complex relationships, nonlinear regression techniques were subsequently applied. These included fitting the data to polynomial and exponential models, which were evaluated for accuracy using standard statistical metrics such as *R* ^2^, root-mean-square error (root mean square error (RMSE)), and mean absolute error (MAE). The performance of the nonlinear models was compared to that of the linear model to assess improvements in the predictive capability.

## Experimental Reproducibility

All experiments were performed in triplicate to ensure reliability and reproducibility. Statistical analyses were conducted to validate the significance of the results, and deviations between replicates were analyzed to evaluate the system’s consistency.

This methodical approach ensured a rigorous assessment of the potential for nonlinear regression to enhance the analytical performance of colorimetric ELISA analysis.

### Machine Learning

Supervised learning refers to a machine learning technique in which an algorithm is trained on labeled data to learn and to model the relation between a set of labeled input variables or features, denoted as *x*_*i*_, and their corresponding output values or labels, named *y*_*i*_. The complete collection of these input and output variables forms the data set D = {(*x*_*i*_, *y*_*i*_)}. A subset of this data, {(*x*_*i*_, *y*_*i*_), *I* = 1, 2, ... *m*}, is referred to as the training set. It consists of m training examples (*x*_*i*_, *y*_*i*_), and it is used to train the model. The goal or aim is for the model to accurately predict and map outputs for new, unseen, and unlabeled data from real-world inputs, with the predicted values falling within one of two categories: classification or regression for sorting into predefined discrete classes or for efficiently and robustly predicting continuous numerical values, respectively.^15^ Regression models and establishes a logical, systematic, and precise mathematical relationship between features and labels, facilitating the generation of reasonably accurate predictions according to the established connection. Moreover, regression models can be either linear or nonlinear. Linear regression, as a comprehensible, rudimentary model, not only builds a transparent and explainable relationship between independent features *x*_i_ and dependent target variables *y*_i_, but also functions as a key indicator and an essential benchmark for evaluating and analyzing the predictive accuracy of more complex and advanced models.^13,16^

In our previous study,^13^ linear regression was employed as the primary machine learning algorithm to predict results comparable to those obtained from FDA-certified clinical plate readers, “Tecan” and “Epoch”. The prediction accuracies were 93.580%, 97.580%, and 97.288% for the HE4, PC3, and 5637 tests, respectively. In exactly the same manner as in earlier work, the data set is constructed from RGB (i.e., RGB) values extracted from the images obtained from microplate wells of ELISA readers. The initial input matrix, referred to as the training set matrix, comprises these three distinct color channels as features, derived from captured images of 72 cancer cell lines as the sample set. Each RGB triplet constitutes a training example, training vector, or stack, and collectively, these stacks construct and form the entire training set matrix *X*. Henceforth, each feature vector of the feature matrix (*X*) represents a single sample or, termed alternatively, one single microplate well. Given the 72 filled wells or training examples, indexed as 1 ≤ *i* ≤ 72, and three-color channels as features, labeled 1 ≤ *j* ≤ 3, adjusted by −1 to align with the zero-based indexing system of Python, *X*_*i*,*j*_ denotes the value of the *i*th training example for the *j*th feature. The corresponding output values are the optical densities (OD) associated with each image.^13^

In the current study, the same data set is used with a more advanced machine learning algorithm, extreme gradient boosting (i.e., XGBoost), to predict and model nonlinear relationships in order to improve precision, accuracy, overall performance, and total success rate.

### Data Set and Feature Engineering

The same preprocessed data set was employed in the XGBoost model; however, additional feature engineering was performed to enhance the model’s capacity to recognize patterns. Three variables, the intensities of RGB, were obtained from images of prostate cancer cell lines captured by a smartphone camera as sample images to be used as input features for the model, with each containing its own RGB values, represented as R (r, g, b), G (r, g, b), and B (r, g, b). In the initial model, regression was applied to estimate the optical density of different prostate cancer cell lines.

In order to enhance the model’s capability, the feature space was expanded from the original three channels by deriving new features from the initial three features or RGB inputs by utilizing a transformation formula. Any of the resulting features consisting entirely of null vectors or all-zero entries were excluded from the data set. Subsequently, this process expands the feature set from three to 12, hence amplifying the model’s expressive power and improving its ability to discover underlying nonlinear patterns more proficiently.

Moreover, in the current research, two different feature expanding was implemented, one the same as the research preceding this and the second one, an improved expansion, with different formulas.

### Extreme Gradient Boosting

Ensemble learning techniques harness the collective strengths of simpler models, defined as base or weak learners, to elevate the model performance to exceed that of any single weak learner. Subsequently, Boosting is a specific method within ensemble learning in machine learning, constructing models by iteratively combining multiple weak learners, evolving them into a powerful model or strong learner, hence minimizing errors and amplifying accuracy. These weak learners progressively develop and yield more precise results by being strategically selected via the boosting algorithm, while each weak learner on its own exhibits a constrained prediction performance. In other words, boosting algorithms can also be viewed as techniques that sequentially fit dynamic basis-functions to the data, thereby resulting in enhanced flexibility and increased model performance, as well as refining and enhancing weak learners by continuously adjusting them to generate superior ones.^17,18^

Gradient boosting, based on the concept of gradient descent in a mathematical space where each point represents a function, or function space, where the model iteratively minimizes a specified loss function by fitting new weak learners to the residuals, is also another ensemble supervised machine learning technique employed excessively for both classifications and regression tasks, creating complex predictive models by combining numerous weak learners, one of which typically is decision trees as base learners. Furthermore, when decision trees are utilized, gradient boosting demonstrates an exceptional empirical and practical performance. The trees are constructed one at a time, with each new tree aiming to correct the residuals of the preceding ensemble, resulting in additive tree models. The residuals are the differences between current predictions and actual labels or target values. The additive nature of the model allows for a precise estimation by progressively refining the prediction. To prevent overfitting and control model complexity, specifically with respect to decision trees, depth, and branches of trees or nodes, regularization techniques, for instance, L1 and L2, are commonly employed. Gradient boosting effectively transforms weak prediction rules into a powerful, accurate model by iteratively reducing errors, trading off increased computational cost and processing for improved accuracy. In brief, for accurate estimation, gradient boosting iteratively combines weak learners through the minimization of loss functions within a cumulative modeling framework, updating the model each time to drive its output closer to the label values.^18–24^

“eXtreme Gradient Boosting” generally known as “XGboost” is a learning framework originally introduced by Chen and Guestrin, which is well recognized among the leading, most widely used, effective, and powerful machine learning algorithms and is rooted in the principles of ensemble learning, gradient boosting, gradient boosted decision trees (GBDT), supervised machine learning, and decision tree methods. XGBoost employs a learning algorithm referred to as “Classification and Regression Trees” denoted as CART. XGboost is renowned for its outstanding speed and performance, having frequently outperformed other models in numerous machine learning competitions.^14,25–27^

Moreover, XGBoost incorporates regularization parameters to mitigate and lessen overfitting, a common obstacle and challenge in gradient boosting methods. The algorithm’s optimized cache utilization, in addition to its support for out-of-core computation, enables significantly faster processing, particularly when handling large data sets. Moreover, despite the inherently sequential nature of boosting, XGBoost incorporates parallelization within its computations, efficiently exploiting all available CPU cores, although it still maintains sequential implementation at the tree level.

Furthermore, XGBoost carries out parallel-tree boosting, automatically handles missing values, and limits tree growth through depth-based pruning, limiting, and restricting growth beyond a certain level. It grows trees in a depth-first technique, in contrast to the levelwise method employed in gradient boosting algorithms. Additionally, XGBoost is renowned for being extremely portable, scalable, accurate, flexible, and efficient. It is used in multiplatforms and is widely applicable in both classification and regularization tasks.

XGBoost is heavily adopted and utilized in scientific data analysis and is also an essential part of the production systems and pipelines of leading technology organizations. Moreover, considering its efficient and optimal employment of computational resources, processing capabilities, and limited demand for system capacity, cache, or memory during the model training phase, XGBoost demonstrates top-notch scalability, making it effective in handling complex, large-scale, real-world machine learning applications, even up to and including those involving deep-learning problems.

XGBoost advances beyond and surpasses traditional boosting tree models and gradient boosting primarily due to the fact that it employs both the gradient and the Hessian (i.e., first and second derivatives) of the loss function by applying an approximation of the loss, based on a second-order Taylor expansion, during the optimization of the objective function with respect to the model’s predictions. The accelerated convergence, precise and minimally biased updates, and superior predictive accuracy of XGBoost over traditional gradient boosting originate from the fact that, while traditional gradient boosting relies solely on gradients or first-order derivatives for model optimization, XGBoost, akin to Newton–Raphson optimization, advances this technique by utilizing both the first- and second-order derivatives. In the functional space, each iteration of boosting corresponds to adding a new decision tree, which models the gradient of the loss function; since each tree depends on the previous one, or termed differently, the sequential nature of boosting, these trees cannot be trained independently. More precisely, while training the tree constructed at iteration *n*, the model depends on the accumulated pseudoresiduals from the preceding *n* – 1 prior trees, making full parallelization of tree construction across iterations particularly complex. Instead, XGBoost optimizes parallelism within the construction of a single tree by simultaneously assessing split point candidates across features, enabling efficient parallel and distributed computing instead of across trees. Moreover, XGBoost demonstrates each data point with a “Gradient–Hessian” duo, stored in two 32-bit floating-point format or float32 to optimize memory usage, while all computation steps, such as summation of gradients and Hessians during split assessments, are executed by utilizing higher precision 64-bit or *float64* double-precision arithmetic for minimizing, lessening, or diminishing numerical and computational errors that may be attributed to floating-point summation, mainly in large data sets or trees where such small errors can accumulate considerably. This leads to overcoming and lowering rounding errors and maintaining accurate split assessment and score or gain calculations. Subsequently, this method equilibrates efficient memory utilization, as well as computational precision. XGBoost incorporates parallel and distributed computing, which results in acceleration in training by performing computations concurrently within individual trees and subsets of data, even though the overall process remains sequential. Furthermore, XGBoost employs processor multithreading, a CPU ability or feature that divides a process or program into multiple minor instructions or tasks called threads, thus leading to parallel execution. Parallelization occurs owing to these threads running in parallel, simultaneously, or concurrently across multiple CPU cores, keeping various processor cores in a chip multiprocessor (CMP) busy with handling tasks and allowing a program or process to carry out many processes at the same time. Since XGBoost automatically executes CPU multithreading capabilities, it constructs tree nodes in parallel and computes gradients for several data points at the same time on different processor cores, allowing it to outperform traditional boosting methods. In addition, XGBoost also utilizes randomization techniques, random sampling of data subsets during training, to diminish overfitting and to speed up the training process by reducing the volume of computational data.^14,17,25,27–33^

In this study, the data set was derived from images captured at three distinct wavelengths corresponding to RGB, generated by a TFT LCD that uniformly illuminated a 96-well ELISA reader microplate using a custom 3D-printed optomechanical setup and a smartphone camera. Given that each wavelength image was obtained from the same filled sample well and contained its own RGB subcolors, the RGB values from each well across the three wavelength images were initially summed accordingly. Furthermore, during postsummation of each RGB wavelength image, three input features depicting the RGB or RGB color intensities were extracted. These RGBs, which correspond to the average pixel intensity within the circular region of each well as seen from a top-down view, where the smartphone was located, function as input features for further computations and analyses. Moreover, these experiments were carried out under identical experimental protocols on four different prostate cancer cell lines, PC3, HE4, 5637, and ACHN, with sample sizes varying across cell lines. For instance, the HE4-cell line data set contained 72 training sets or samples. The data set can generally be defined as follows

$$D=\left\{(x_{i},y_{i})\right\}\left(\left|D\right|=n,\;x_{i}\in R^{m},y_{i}\in R\right)$$

where each *x*_*i*_ represents the input features, with each RGB-trio or training example denoted as *x*_*i*_, and *y*_*i*_ is the corresponding label acquired from FDA-certified and commercially available clinical plate readers, named EPOCH and Tecan. Furthermore, for the HE4 cell line, *m* = 72 denotes the number of training sets, and *n* initially equals 3. Figure 2a–c illustrates the input values.

The preliminary three features were expanded through feature engineering by applying a formulated transformation function on the original RGBs in order to construct new and additional features, and for each generated feature, another transformation formula was implemented. To confirm that the new features were effective, practical, relevant, and valid, all entire-zero valued columns were eliminated, resulting in the expansion of the feature space from the initial 3 channels to a total of 12 features. Consequently, the heatmap visualized in Figure 2d represents the resulting feature space or the complete training set, comprising 72 data instances as well as 12 features, with each row corresponding to one training example.

**Figure 2:**
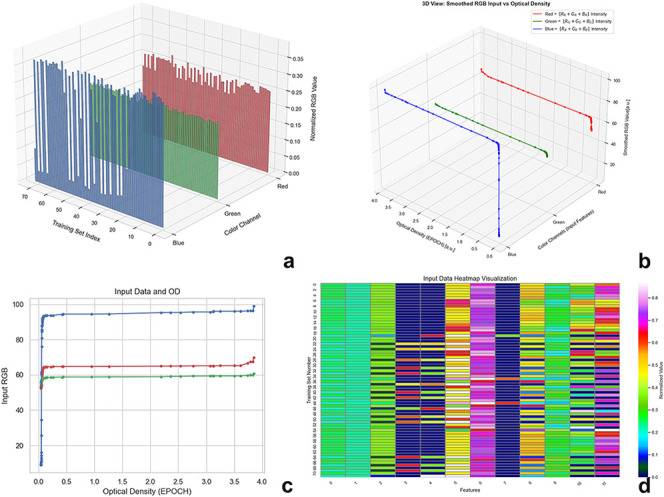
Input data and their corresponding optical densities represented in different types (a–c,d) demonstrate HE4 cell line inputs post feature engineering, where the features were expanded from 3 to 12.

In regression tree ensemble models, the predicted output for the *i*th training example, *ŷ*_*i*_, given input *x*_*i*_, is built by the summation of the outputs of several regression trees, or additive functions, denoted by *f*_*k*_(*x*) and indexed by *k*. Each *f*_*k*_(*x*) presents a regression tree function employed on input *x*_*i*_. To put it differently, the predicted output for the *i*th training example is as eq 1 below

1 $${ŷ}_{i}=\varphi \left(x_{i}\right)=\sum_{k=1}^{K}f_{k}\left(x_{i}\right),,f_{k}\in F$$

where F is expressed as the space of all possible regression trees or CARTs that the model can be built from; in other words, at every boosting iteration or *k*, *f*_*k*_ is selected from the function space defined as

$$F=\left\{f\left(x\right)=w_{q\left(x\right)}\right\},\left(q:R^{n}\to T,w\in R^{T}\right)$$

Furthermore, *f*(*x*), the prediction function, is given by *w*_*q*(*x*)_, where each *w* refers to scores or values corresponding to each leaf node in a decision tree and *w*_*i*_ represents *i*th-leafs’ score; in other words, it is the score of the leaf node chosen by *q*(*X*). Additionally, *q*(*x*), the function structure of a tree, maps a feature vector $x\in R^{n}$ , specifically *n* = 12 in this research, to an explicit leaf index, (1, 2, 3, ..., *T*), *T* operating as the total number of leaf nodes. This leaf index presents the position of the leaf node that *x* arrives at after traveling through the decision tree. Furthermore, *q*(*x*) establishes the route that a feature vector *x* follows within the decision tree to reach a targeted node.

The Objective function in XGBoost or gradient-boosted trees is defined as

2 $$L\left(\varphi \right)=\sum_{i}l\left({ŷ}_{i},y_{i}\right)+\sum_{k}\Omega \left(f_{k}\right)\Omega \left(f\right)=\gamma T+\frac{1}{2}\lambda {∥w∥}^{2}$$

where the first term *l*(*y*_*i*_,*y̅*_*i*_) is termed as the convex loss function, which is also differentiable, measuring the difference between the predicted value and the label, *y̅*_*i*_ and *y*_*i*_, respectively. Subsequently, the second term, Ω, is the penalization or regularization term introduced to penalize and control the model complexity and prevent overfitting by limiting the number of leaves. Furthermore, γ*T* is the penalty for *T* per decision tree, restricting excessive tree structure complexity. In addition, the second term in Ω, 1/2λ||*w*||^2^, is the expression applied for L2 regularization to *w,* where λ is the regularization parameter. This provides a trade-off between predictive accuracy, denoted by *l*(*y*_*i*_,*y̅*_*i*_) and model complexity, or Ω.^14,25^

During each iteration t of the boosting procedure, XGBoost optimizes the objective function in eq 3 by utilizing a second-order Taylor expansion

3 $$L^{\left(t\right)}=\sum_{i=1}^{n}l\left(y_{i},{ŷ}_{i}^{\left(t-1\right)}+f_{t}\left(x_{i}\right)\right)+\Omega \left(f_{t}\right)$$

4 $$\begin{array}{l} L^{\left(t\right)}\approx \sum_{i=1}^{n}\left[l\left(y_{i},{ŷ}_{i}^{\left(t-1\right)}\right)+g_{i}f_{t}\left(x_{i}\right)+\frac{1}{2}h_{i}{f_{t}}^{2}\left(x_{i}\right)\right]+\Omega \left(f_{t}\right)\\ g_{i}=\frac{\partial l\left(y_{i},{ŷ}_{i}^{\left(t-1\right)}\right)}{\partial {ŷ}_{i}^{\left(t-1\right)}}\\ h_{i}=\frac{\partial^{2}l\left(y_{i},{ŷ}_{i}^{\left(t-1\right)}\right)}{\partial {ŷ}_{i}^{{\left(t-1\right)}^{2}}} \end{array}$$

5 $${\mathrm{L̃}}^{\left(t\right)}=\sum_{i=1}^{n}\left[g_{i}f_{t}\left(x_{i}\right)+\frac{1}{2}h_{i}{f_{t}}^{2}\left(x_{i}\right)\right]+\Omega \left(f_{t}\right)$$

where at the *t*th update, the model learns *f*_*t*_(*x*_*i*_) regression tree in the boosting process. In order to identify and determine the optimal and most efficient *f*_*t*_ that minimizes L , both the Hessian *h*_*i*_ and the Gradient *g*_*i*_ of the loss function are utilized, enabling the model to incorporate the regression tree *f*_*t*_, built by employing a greedy algorithm, selecting the most-effective splits according to the gain-score acquired from eq 6, thereby efficiently improving the objective L . Gain-score is the quantified enhancement in the model’s predictive accuracy resulting from the utilization of a distinct feature at a given split, or in other words, the proportional impact of a given feature on the performance, which should be maximized to improve the model. Subsequently, gain is expressed as

6 $$Gain=\frac{1}{2}\left[\frac{{\left(\sum_{i\in I_{L}}g_{i}\right)}^{2}}{\sum_{i\in I_{L}}h_{i}+\lambda }+\frac{{\left(\sum_{i\in I_{R}}g_{i}\right)}^{2}}{\sum_{i\in I_{R}}h_{i}+\lambda }+\frac{{\left(\sum_{i\in I}g_{i}\right)}^{2}}{\sum_{i\in I}h_{i}+\lambda }\right]-\gamma$$

where γ represents the minimum gain threshold required to permit a split and control tree growth, and *I*_L_ and *I*_R_ are the left and right instances, respectively. Furthermore, gain is a compromise between the first term, or the reduction in training loss, and the regularization penalty term; as can be seen, gain can have a negative value. When this occurs, the tree continues to grow to its predefined maximum depth or until termination conditions are satisfied, as subsequent splits may compensate for earlier negative gains. Afterward, postpruning is deployed to remove splits with negative gain, thereby preventing further splitting of those nodes. Moreover, the leaves are only allowed to split if the gain exceeds the threshold, functioning as the prepruning technique.^14,17,25–27^

## Results and Discussion

In this study, the XGBoost regression model was executed with *k* = 100, with a maximum tree depth of 4, and 0.1 as the learning rate. Furthermore, 80% of the data set was allocated for training, while the remaining 20% subsamples were utilized for testing. The model was done utilizing the XGBoost Python library.

To assess the efficiency and performance of the XGBoost model, the metrics in Table 1 were calculated and compared with those of the corresponding linear regression model.

**Table 1:**
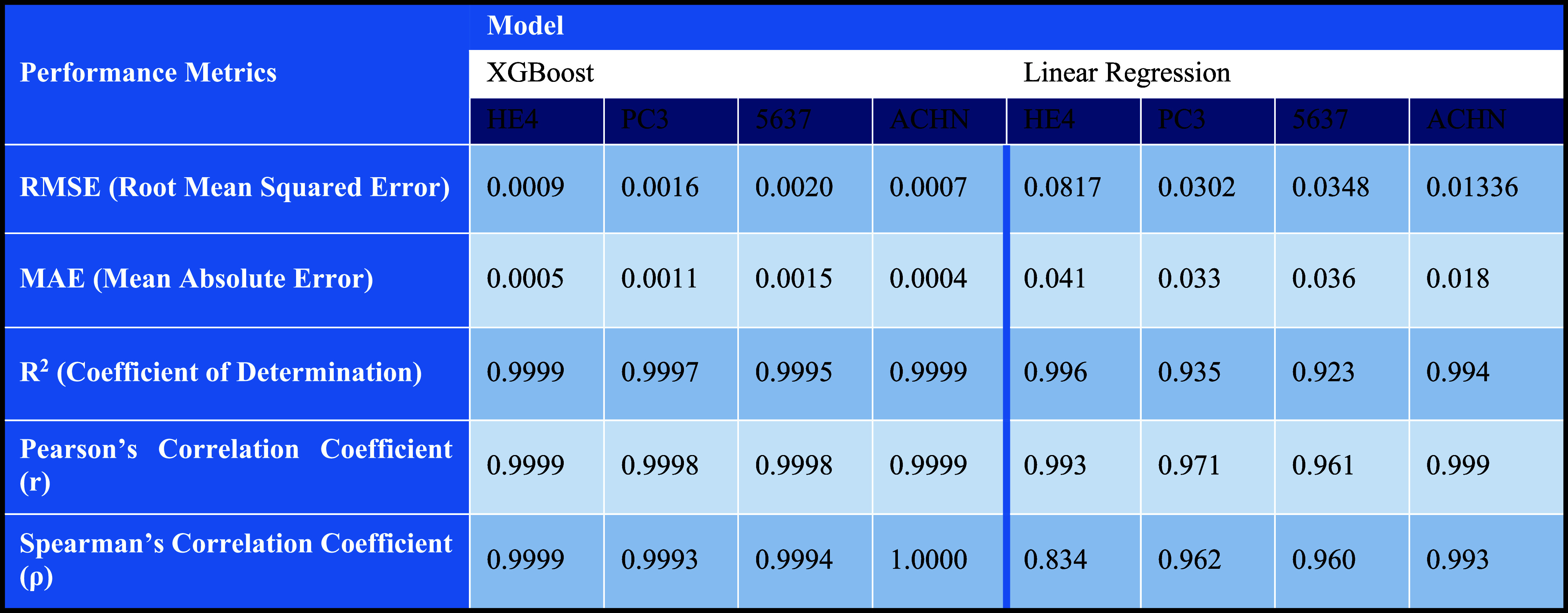
Comparison of XGBoost and Linear Regression Models in Optical Density Prediction Performance

To evaluate the reliability and predictive accuracy of the model’s outputs, famous statistical metrics were calculated. The main objectives were to minimize the root-mean-square error (RMSE) and MAE and to maximize the coefficient of determination (*R* ^2^). RMSE was calculated as eq 7 for both the training and testing data sets. RMSE is particularly responsive to outliers and effectively captures the difference between actual labels and the predictions. Furthermore, MAE is the absolute mean of the differences between the actual Epoch-ODs and the predicted values or errors. The closer RMSE and MAE are to 0, the more accurate the outputs are. Subsequently, *R* ^2^, with values ranging between 0 and 1, measures how effectively the model captures the relation between the RGB intensities and the actual Epoch-ODs. It defines the ratio of the total variation in Epoch-OD that can be predicted from the inputs. On the one hand, if *R* ^2^ = 1, it indicates an exact match; in other words, the predictions ideally match the Epoch-ODs, and variations in the inputs are reflected in the outputs. On the other hand, if the value is 0, the model fails to describe variations in the data.^30^

7 $$RMSE=\sqrt{\frac{1}{n}{\left(y^{\left(i\right)}-ŷ\right)}^{2}}$$

8 $$MAE=\frac{1}{n}\sum_{i=1}^{n}\left|y^{\left(i\right)}-\hat{y_{i}\;}\right|$$

9 $$R^{2}=1-\frac{RSS}{TSS}=1-\frac{\sum_{i=1}^{n}{\left(y^{\left(i\right)}-ŷ\right)}^{2}}{\sum_{i=1}^{n}{\left(y^{\left(i\right)}-\mathrm{y̅}\right)}^{2}}$$

where RSS is the residual sum of squares, TSS is the total sum of squares, and *y̅* is the average of *y*.

Two other metrics, Pearson’s correlation coefficient (r) and Spearman’s correlation coefficient (ρ), were also applied. Primarily, *r*, which ranged between −1 and 1, demonstrates the quantification of the strength of the linear correlation between the output values and the Epoch-OD. In the event that *r* = ± 1, there is a perfect linear correlation, and with *r* = 0, there is no linearity. Furthermore, ρ assesses the ranks rather than values, meaning it indicates whether an incrementation in one variable corresponds to a constant increase or decrease in the other variable or evaluates the trends. Meanwhile, if ρ = ± 1, there is an exact increase or decrease between the variables, while ρ = 0 means no consistent trend between the two variables.

The RMSE, MAE, *R* ^2^, Pearson’s coefficient, and Spearman’s coefficient data evidently demonstrate the exceptional XGBoost regression model’s proficiency in predicting the optical densities as outputs from RGB intensities as input features and validate the model’s reliability, precision, and accuracy in this process. To analyze more deeply, the 0.0009 RMSE and 0.0005 MAE trigger outstanding accuracy and precision in prediction. The *R* ^2^ value yields an exceptionally high value of 0.9999, indicating that 99.99% of the variance in the Epoch-OD is defined by RGB inputs. Moreover, Pearson’s and Spearman’s correlation coefficients are calculated as 0.9999, meaning an approximately perfect linear relation, as well as the predictions following the RGB values’ trend. Although XGBoost, an inherently nonlinear tree algorithm, was employed, the predictions proved exceptionally high Pearson and Spearman correlation coefficients, meaning the relation between the prediction and the Epoch-OD is linear, and the model effectively captures nonlinearity and transforms it into a space where the predictions align linearly with the epoch-OD.

The results clearly indicate that the performance significantly surpasses and exceeds that of previous papers’ linear regression model, with an RMSE of 0.0817. This leads to an improved and more refined model with enhanced and precise prediction accuracies, paving the way for future research to classify the results according to the cell line types.

Figure 3 demonstrates the logarithmic relation between XGBoost-predicted values and the actual Epoch-OD values on the *Y*-axis and *X*-axis, sequentially. The relatively close tracking between the Log­((Epoch-OD), Log­(Epoch-OD)) diagonal line to the models’ predictions (Log­(Epoch-OD), Log­(XGBoost-OD)) sheds light on the model’s accuracy, robustness, reliability, and strength.

**Figure 3:**
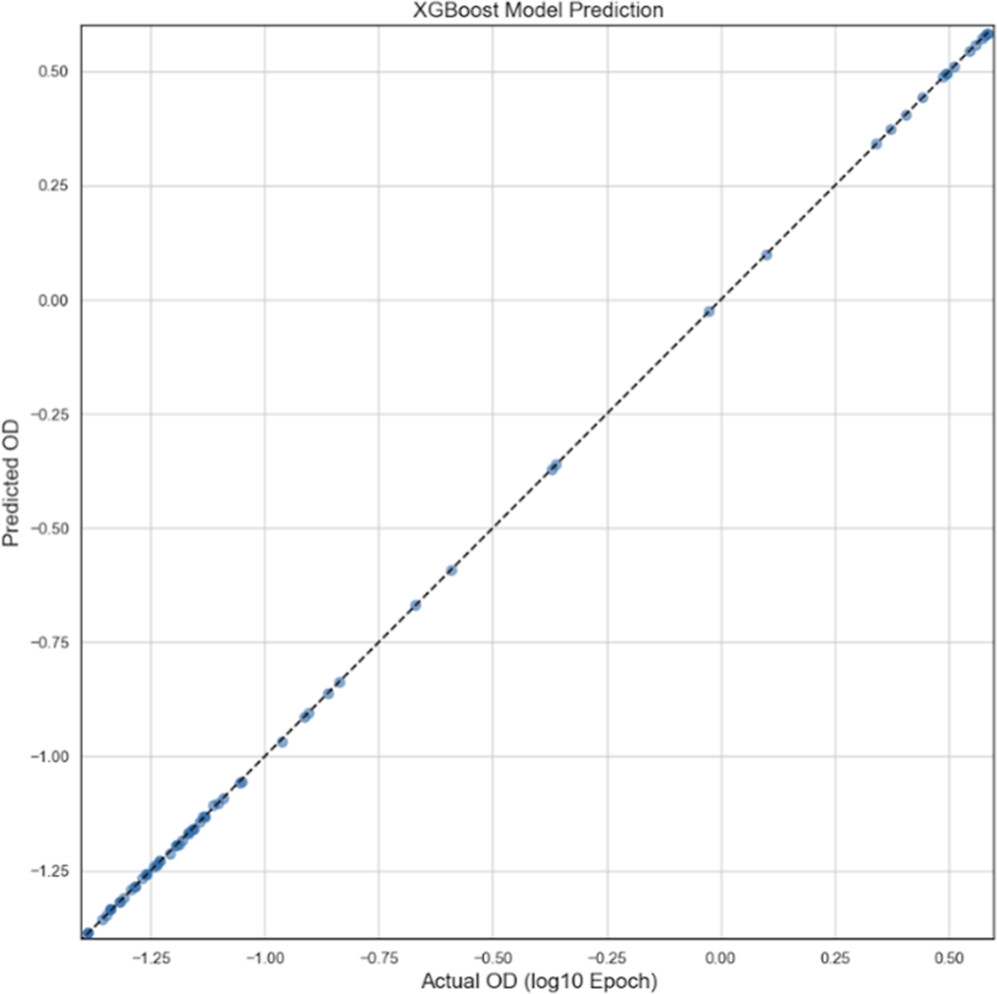
Predicted optical densities vs epoch optical densities displayed on the logarithmic scale.

In comparison to previous studies summarized in Table 2 that utilized paper strips or reported sensitivity values between 72 and 89.5%, classification accuracies around ∼99%, or linear calibration with *R* ^2^ = 0.9994, the same device exhibits superior quantitative accuracy by enabling nonlinear XGBoost based prediction of optical density (OD) with a >99% reduction in RMSE compared to our previous linear model and Pearson and Spearman correlation coefficients close to unity. This performance is achieved while maintaining eco-friendliness due to the absence of third-party optical attachments and its reusability. Furthermore, its direct utilization of smartphone-captured RGB images, without the need for labeling with nanoparticles or other materials, provides a competitive advantage over similar systems with comparable objectives. The absence of third-party involvement allowed each 96-well plate to accommodate different biomarkers or cancer cell lines, enabling simultaneous detection of every well. Cost-effectiveness remains a major advantage of this system.

**Table 2:** Comparative Performance and Features of the Smartphone-Based Optical Device and Previous Studies

reported performance	0.923–0.996	mare antibodies: LOD = 1.72 ng/mL (smartphone) vs 1.14 ng/mL	>99%	75.7% detection vs 18.9% by conventional ELISA	86.05% detection; AUC = 0.96	0.9994	LOD = 19 pg/mL (using B channel for calibration)
reported metric	*R* ^2^	LOD	classification accuracy	detection sensitivity	detection rate/AUC	*R* ^2^	LOD
assay format	standard 96-well ELISA	96-well microplates (protein assays + immunoassay)	ELISA	immunosensing assay (SARS-CoV-2 NP)	immunosensing (SARS-CoV-2 IgG)	standard 96-well microtiter plate	standard 96-well ELISA (TNFα)
illumination	LCD	smartphone imaging	LED	external/fiber-integrated	external/fiber-integrated	LED (optical attachment)	constant illumination
platform/method	smartphone + linear regression	smartphone optical readout + app image processing	smartphone + ML	smartphone + fiber-integrated immunosensing	smartphone + high-throughput fiber-optic immunosensor	smartphone-based portable 96-well microplate reader + app-based colorimetric analysis	3D-printed DIY 96-well ELISA plate reader
study	Mirhosseini et al., 2024 ^13^	Gómez et al., 2024 ^34^	Berg et al., 2016 ^35^	Wu et al., 2022 ^36^	Wei et al., 2025 ^37^	Deng et al., 2023 ^38^	Pohanka et al., 2024 ^39^

## Conclusion

In order to continue the previous research, which modeled the input RGB intensities to FDA-approved benchtop ELISA reader Epoch-ODs with linear regression, in the current study, a nonlinear algorithm, XGBoost, was utilized. XGBoost outperforms linear regression and is excellent in modeling complex and nonlinear relations and patterns. Furthermore, it enhances the accuracy and performance of the portable smartphone-based 3D-printed optomechanical device functioning as an ELISA reader, making it a strong candidate for smartphone-based diagnostics. This results in improved performance and enhanced prediction with various samples and different smartphones, even in low-light conditions. Furthermore, as opposed to linear regression, which expects a linear relation between input variables and the predicted output, XGBoost utilizes several decision trees to capture nonlinear relations by iteratively minimizing the loss function. XGBoost pushes the model’s performance beyond the linear regression model, with 99.946%, 99.885%, 99.843%, and 99.959% accuracy compared to 93.580%, 97.580%, 97.288%, and 98.993% for the HE4, PC3, 5637, and ACHN tests, respectively. Future work should focus on classifying the cell lines, validating the model with different data sets beyond cell lines, and exploring the device’s applicability in real-time scenarios.

## Abbreviations

XGBoost: eXtreme Gradient Boosting
RGB: Red, Green, and Blue
ELISA: enzyme-linked immunosorbent assay
LFA: lateral flow assays
