# Correction to “Advancing Colorimetric Analysis in Enzyme-Linked Immunosorbent Assays: Harnessing Nonlinear Regression for Improved Accuracy and Predictive Performance”

**DOI:** 10.1021/acsomega.6c07928

**Published:** 2026-08-21

**Authors:** Shaghayegh Mirhosseini, Aryanaz Faghih Nasiri, Fatemeh Khatami, Akram Mirzaei, Seyed Mohammad Kazem Aghamir, Nathan S Swami, Mohammadreza Kolahdouz

The Acknowledgments section of this article incorrectly noted support from NIH grant R01GM154950 for this work. We regret this honest mistake in citing this grant, which was not used to support any portion of this work.

